# Impacts of the COVID-19 Pandemic on Primary Care Utilization: An Analysis of Primary Care Claims Data in Alberta, Canada

**DOI:** 10.1177/21501319251338376

**Published:** 2025-06-17

**Authors:** Mina M. Fahim, Richard P. Golonka, Robin L. Walker, Alka B. Patel, Mary V. Modayil, Lisa L. Cook, John Hagens, Rob Skrypnek, Judy Seidel

**Affiliations:** 1 Primary Care Alberta, AB, Canada; 2 University of Calgary, AB, Canada; 3 University of Lethbridge, AB, Canada; 4 Alberta Health Services, AB, Canada

**Keywords:** COVID-19, epidemiology, health services research, primary care

## Abstract

**Background:**

The COVID-19 pandemic disrupted primary health care systems worldwide, prompting rapid changes in how care was delivered. In Alberta, this included a significant shift from in-person to virtual care. This study examines trends in primary care utilization among Albertans during COVID-19 and the shift toward virtual care.

**Methods:**

Repeated cross-sectional analyses were conducted from 2018/19 to 2022/23 using Alberta Health Practitioner Claims data. Utilization was measured as the proportion of Albertans with at least one visit and the annual visit rate per person. Annual percent change (APC) was calculated relative to the pre-pandemic year (2019/20) and stratified by demographics.

**Findings:**

The proportion of Albertans with a primary care visit decreased by −9.55% in 2020/21 but recovered to −4.62% by 2022/23. Annual visit rates remained stable post-pandemic. The largest declines in 2020/21 were among children aged 5 to 11 (−38.42%), ≤4 (−33.42%), newborns (−30.36% to −25.49%), and those without health conditions (−20.9%). Virtual care accounted for 23.77% of visits in 2020/21, dropping to 14.43% by 2022/23.

**Conclusions:**

While fewer Albertans accessed primary care, visit rates remained stable due to virtual care. Further research is needed to assess the long-term impacts of COVID-19 on primary healthcare delivery.

## Introduction

Primary health care (PHC) provides a pivotal access point for care, early intervention, and the coordination of healthcare and services in a community.^1,2^ A well-integrated PHC system offers several benefits, including continuity of care, the ability to identify and address upstream factors influencing health, and system-level improvements that streamline operations and reduce costs.^3,4^ In Alberta, the advantages of this integration became evident during the COVID-19 pandemic with the introduction of virtual care billing codes and widespread distribution of personal protective equipment to clinics.^5^ Alberta’s COVID-19 Integrated Pathway program further expedited integration by linking unattached patients to a family physician, facilitated rapid sharing of COVID-19 test results, and the development of clinical algorithms to support family physicians in delivering timely care to patients with COVID-19.^6^ Despite these achievements, disruptions to normal patient interactions with PHC has been observed among Albertans, the extent which remains unclear.

Health care systems globally have reported substantial reductions in volume of in-person primary care visits immediately following the onset of COVID-19, concomitant with the rapid rollout and adoption of virtual care.^7-11^ A multi-national analysis conducted by the INTernational ConsoRtium of Primary Care BIg Data Researchers (INTRePID) revealed that countries implementing virtual care managed to counteract declining rates of in-person visits, while countries that did not adopt virtual care experienced sustained decreases in overall visit throughout the pandemic.^7^ The initial decline in primary care utilization mainly stemmed from widespread fear and anxiety associated with COVID-19 infection risks, coupled with temporary or permanent closures of primary care clinics due to reduced visits and a higher-than-normal rate of physician retirements during the pandemic.^5,12-16^ Furthermore, the rapid emergence of virtual care lends the need to explore how various demographics are adopting this modality of care to better identify potential barriers. Additionally, the long-term recovery of primary care utilization several years following the onset of COVID-19 has yet to be explored.

Therefore, the objectives of this study are to (1) compare primary care utilization pre-pandemic to 1- to 3-years post-pandemic for Albertans at a population level, and (2) describe trends in the adoption of virtual care. Understanding the changes to primary care utilization and shifts to virtual care at a population level may serve as an indicator of evolving patient health needs and inform health services planning at a provincial level.

## Methods

### Study Setting and Design

Alberta is a western Canadian province with over 4.4 million residents, characterized by a mix of urban centres, rural communities, and remote areas. The provincial health system is publicly funded and administered by Alberta Health Services (AHS), which operates across 5 geographic zones: Calgary, Edmonton, Central, North, and South. Socioeconomic conditions vary widely across the province, with disparities in health access and outcomes observed across geographic and deprivation levels. Alberta has a single-payer health insurance plan (AHCIP) that covers medically necessary physician services for eligible residents.

We conducted repeated cross-sectional analyses to describe primary care utilization in Alberta, Canada, for each fiscal year (FY) from April 1, 2018, to March 31, 2023. Since COVID-19 began near the end of FY 2019/20 (March 11, 2020), this period was designated as one-year pre-pandemic and used as a reference for comparisons.^17^ Virtual care utilization was analyzed from FYs 2020/21 to 2022/23 following the introduction of virtual billing codes. We examined population demographics and clinical characteristics to identify subpopulations with the greatest changes in utilization.

### Participants

All Alberta residents with active AHCIP coverage between FYs 2018/19 and 2022/23 were included. The AHCIP provides coverage for medically necessary physician services. Non-eligible residents include Canadian Armed Forces members, federal penitentiary inmates, refugee claimants, and individuals with expired immigration documents.

### Variables

Demographic characteristics included sex (female, male), age group (≤4, 5-11, 12-17, 18-24, 25-39, 40-64, 65-79, ≥80), geographic location (remote, rural, urban), provincial zone (Calgary, Central, Edmonton, North, South), and the Pampalon material deprivation index (MDI). Geographic variables were determined using residential postal codes based on Alberta’s Official Standard Geographic Areas.^18^ The rural-urban spectrum was classified as urban (metro, metro-influenced, urban), rural (moderate urban influence, rural, rural center), and remote. The MDI, derived from the Pampalon Deprivation Index (PDI), categorized individuals into 5 quintiles, with Q1 representing the least deprived areas.^19^

Clinical characteristics included the usual provider of care (UPC) index and the Canadian Institute for Health Information (CIHI) health profile group (HPG). The UPC index, measuring care continuity, was calculated by dividing visits to a primary care clinic or provider by total visits over 3 years. Individuals with ≥3 visits in 3 years were assigned a score (0-1). Categories included: no visits (none in 3 years), no index (1-2 visits), low continuity (UPC 0-0.39), moderate (0.40-0.79), and high (≥0.80). CIHI’s case-mix classifications determined HPG categories: Healthy/major newborn, minor/moderate/major acute, minor/moderate/major chronic, major/other cancer, major/other mental health, obstetrics, palliative, and health system user with no conditions.^20^

The primary outcome was primary care visits, categorized by modality (in-person, virtual). In-person visits were those to family physicians in a clinic. Virtual visits were identified using physician billing codes introduced post-COVID-19. Duplicate visits were removed if a patient saw the same provider at the same facility on the same day using the same modality.

### Data Sources

The Alberta Provincial Registry database provided demographic characteristics and the population denominator. Eligible Albertans were linked to health administrative databases using personal health numbers. The Alberta Health Practitioner Claims database contained all fee-for-service and shadow-billed claims. The CIHI Population Health Grouper database provided HPG classifications.

### Analysis

We calculated the proportion of Albertans with ≥1 primary care visit and the annual visit rate per FY. FYs 2018/19 (T-2) and 2019/20 (T-1) were designated as 2- and 1-year pre-pandemic, respectively. FYs 2020/21 (T1), 2021/22 (T2), and 2022/23 (T3) were 1-, 2-, and 3-years post-pandemic, respectively. Each FY was compared to 2019/20 (T-1) using the annual percent change (APC):

$$APC=\frac{end\;rate-initial\;rate}{initial\;rate}*100$$

To illustrate primary care modality changes, we plotted weekly in-person and virtual visit rates from FY 2019/20 to 2022/23. We reported annual virtual visit rates for post-pandemic FYs and virtual utilization as a percentage of all visits:

$$Virtual\;\%=\frac{virtual\;visit\;rates}{all\;visit\;rates}*100$$

We analyzed the APC of virtual visit percentages year-over-year and summarized trends using the average annual percent change (AAPC). Primary care utilization measures were stratified by demographic and clinical characteristics.

## Results

### Proportion of Albertans With At Least One Primary Care Visit

The proportion of Albertans with at least one primary care visit was 69.73% in T-1, dropping to 63.07% in T1, an APC of -9.55% (Table 1). During T2 and T3, the APC in proportion gradually recovered to −5.97% and −4.62%, respectively (Table 1).

**Table 1.** Percent Proportion^a^ of Albertans With a Primary Care Visit and APC Relative to T_−1_.

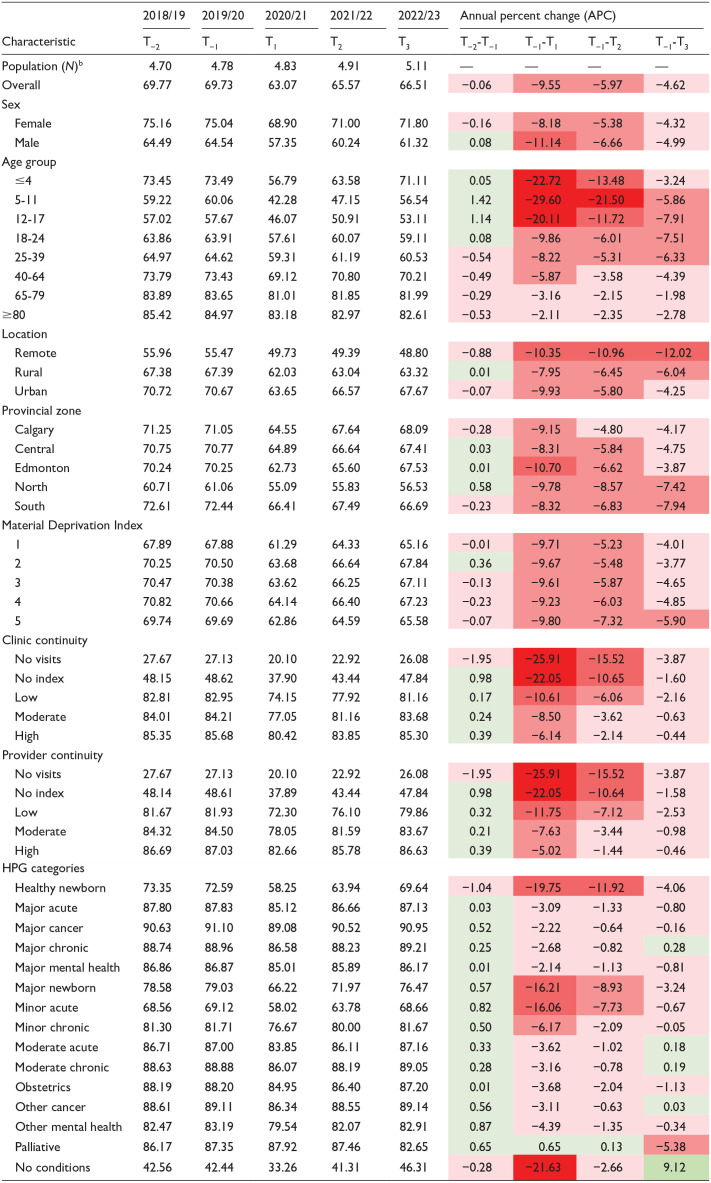

a Percent proportion is calculated as population with at least 1 visit/total population.

b Population is reported per 1 million people.

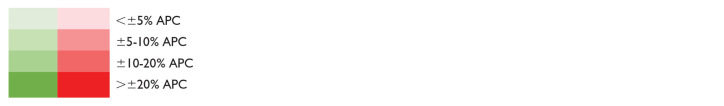

Younger cohorts (<18 years) experienced the largest declines, with 5 to 11-year-olds showing an APC of −29.60% between T-1 and T1 (Table 1). Although all age groups recovered within 10% of pre-pandemic levels, younger cohorts continued to show larger negative APC values in T3 (Table 1). Albertans in remote areas had the lowest proportion of visits and the largest APC decline from T1 to T-1 (−10.35%), which worsened by T3 (−12.02%; Table 1).

Higher continuity of care was associated with smaller APC declines (Table 1). Three years post-pandemic, all continuity groups recovered within 3% of T-1 values (Table 1). Among HPG categories, the greatest declines occurred in health system users with no conditions (−21.63%), healthy newborns (−19.75%), major newborns (−16.21%), and minor acute patients (−16.06%), all of whom recovered within 5% of pre-pandemic levels (Table 1).

### Annual Rates of Primary Care Visits

Primary care visit rates remained stable, ranging from 3.43 in T-2 to 3.57 in T2 (Table 2). A gradient across age groups showed younger cohorts (<18 years) with negative APC values, while older cohorts had positive APCs between T-1 and T1 (Table 2). By T3, all age groups had recovered within 5% of pre-pandemic rates (Table 2).

**Table 2.** Annual Rates^a^ of Primary Care Visits Among Albertans and APC Relative to T_−1_.

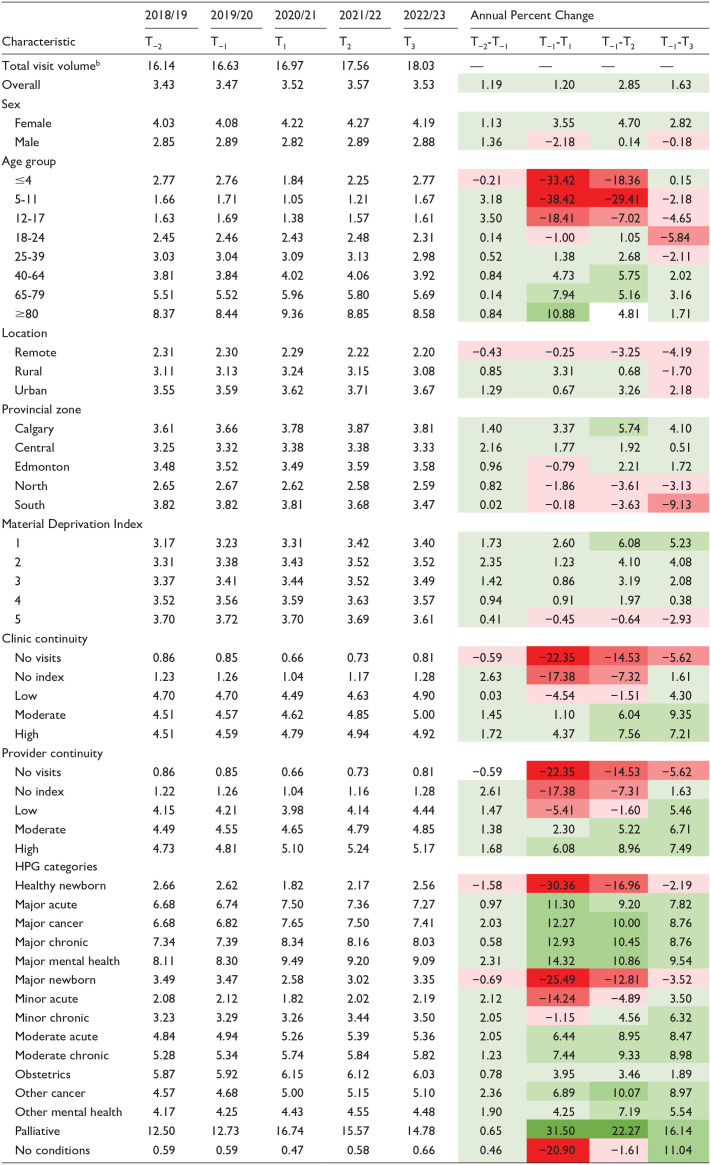

a Annual rates calculated as visit volume/total population.

b Total visit volume is reported per 1 million visits.

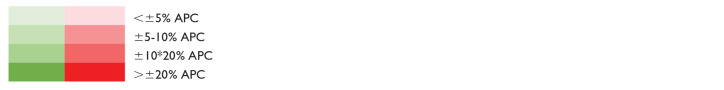

Albertans in remote areas consistently had lower visit rates than rural and urban counterparts, with continued declines in T3 (Table 2). HPG categories with the largest rate decreases between T-1 and T1 included healthy newborns (−30.36%), major newborns (−25.49%), health system users without conditions (−20.90%), and minor acute patients (−14.24%; Table 2). Palliative patients had the highest positive APC (31.50%) (Table 2). By T3, all patient groups returned to pre-pandemic rates except for healthy (−2.19%) and major (−3.52%) newborns (Table 2).

### Shift to virtual primary care

**Figure 1.**
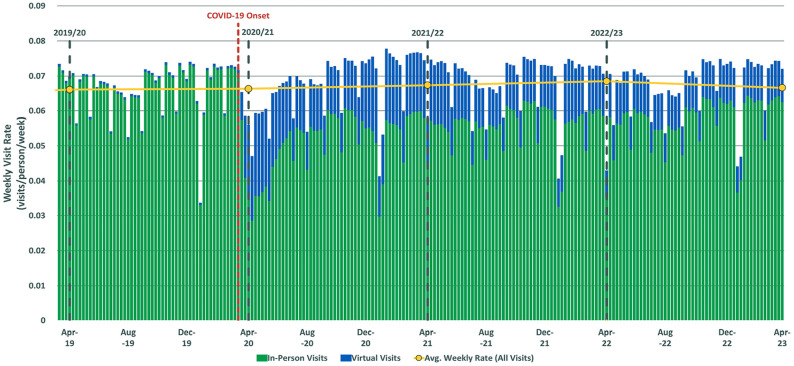
Weekly visit rate of primary care visits in Alberta from 2019/20 to 2022/23.

Before the pandemic, primary care visits in Alberta were almost entirely in-person (Figure 1). COVID-19 led to an immediate drop in total visits and a rapid shift to virtual care (Figure 1).

The annual virtual visit rate was 0.84 in T1, declining to 0.65 in T2 and 0.51 in T3, with an AAPC of −22.07% (Table 3). Utilization was lowest among children ≤4 years (15.16%) and highest among adults 40 to 64 years (25.56%) in T1 (Table 3). Urban residents had the highest virtual care utilization (24.19%), followed by rural (22.23%), and remote (18.89%) populations (Table 3).

**Table 3.** Annual Rates of Virtual Primary Care Visits Among Albertans and AAPC.

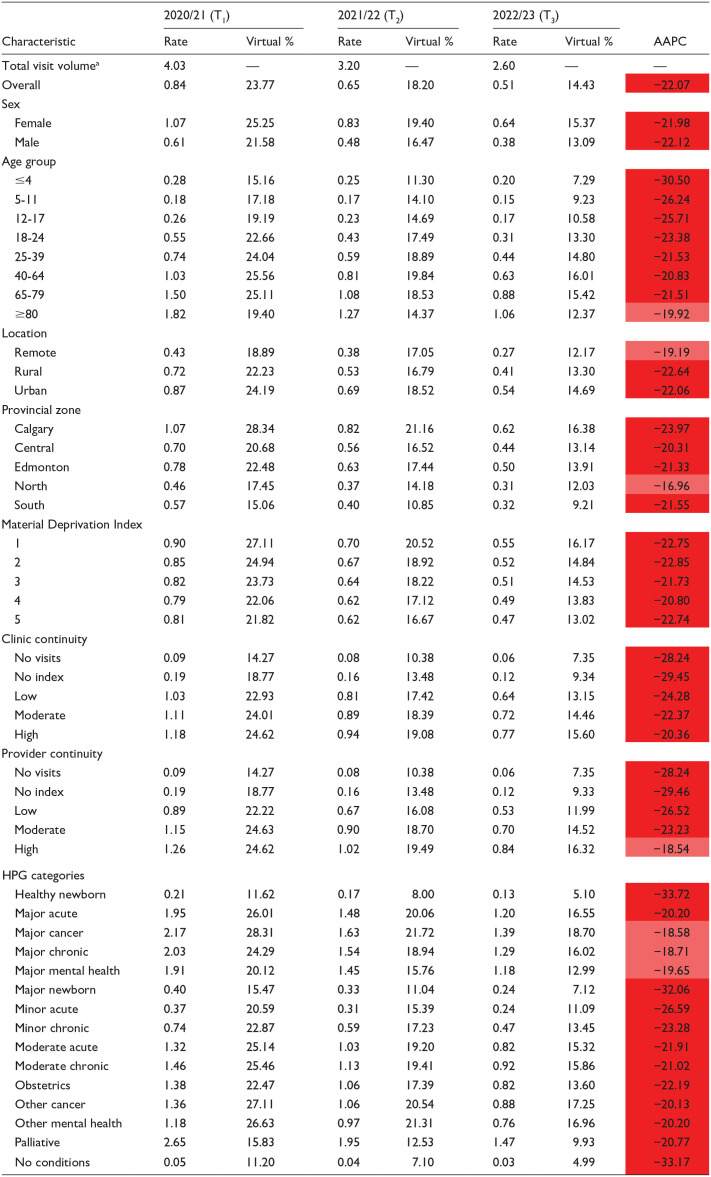

a Total visit volume is reported per one million visits.

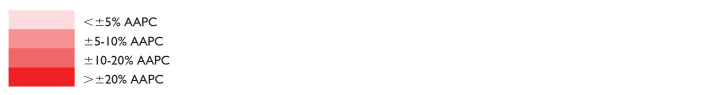

Patients with major or other cancer had the highest virtual care use (28.31% and 27.11%, respectively), while health system users without conditions (11.20%), healthy newborns (11.62%), major newborns (15.47%), and palliative patients (15.83%) had the lowest utilization in T1 (Table 3).

## Discussion

Primary care utilization in Alberta changed significantly during the COVID-19 pandemic. A nearly 10% decrease in proportions compared to a 1% increase in rates 1-year post-pandemic suggests that while fewer Albertans sought primary care, those who did had more frequent visits. The greatest declines in utilization 3 years post-pandemic were observed among individuals aged 5 to 39, those with no or few prior visits, those with lower provider and clinical continuity, residents of the North or South zones, those in rural or remote areas, individuals living in areas with the highest deprivation levels, and those categorized as healthy/major newborns.

During the early pandemic, in-person visits declined sharply, followed by an immediate surge in virtual care (Figure 1). Throughout the post-pandemic years, periods of declining in-person visits coincided with increased virtual care use (Figure 1). While in-person visits have not fully returned to pre-pandemic levels, virtual care adoption helped stabilize overall primary care visit rates. However, virtual care utilization has declined in recent years, raising questions about its sustainability (Table 3). Additionally, some patient groups utilized virtual care less frequently, including males, individuals ≤17 years, those in the North or South zones, those in remote areas, those with high deprivation levels, and those categorized as healthy/major newborn or palliative. Barriers at patient, provider, and systemic levels likely contributed to this variation. For example, limited access to technology, low digital literacy, and inadequate digital infrastructure may have restricted virtual care use for older adults, materially deprived individuals, and those in rural or remote areas.^21^

The steep decline in primary care visits following the onset of COVID-19 significantly disrupted Alberta’s healthcare system and primary care practices. Leslie et al^5^ reported that providers faced severe revenue losses due to reduced in-person visits, partially offset by the introduction of virtual billing codes. Similarly, an Ontario-based study by Glazier et al^8^ found a 28% decrease in total primary care visits within 4 months of COVID-19’s onset compared to the same period a year prior. Their analysis showed that children (<18 years) and individuals with low expected healthcare use experienced the most substantial declines in visits. Findings from the INTRePID analysis in Ontario suggested that recovery in primary care visit rates 1 year post-pandemic was primarily driven by virtual care uptake.^7^ Other research indicates that older and lower-income patients were less likely to adopt virtual care. These findings emphasize the need to implement long-term solutions to overcome barriers to virtual care, such as improving digital infrastructure, addressing provider workflow and system integration challenges, and ensuring sustainable virtual billing models.^11,22,23^ Further evaluation and research should explore how social determinants of health influence virtual care access, particularly within urban and rural populations that have experienced recent declines in use.

This study represents the first comprehensive examination of primary care utilization post-pandemic in a Canadian province outside Ontario and the first to assess recovery several years after COVID-19. A limitation of this analysis is the use of fiscal year reporting, as the onset of COVID-19 (March 11, 2020) overlapped with the final weeks of the “1-year pre-pandemic” period (March 31, 2020). This overlap may have led to the misclassification of T-1 and underestimation in APC calculations.

The shift from in-person visits to virtual care marked a profound transformation in healthcare utilization. While virtual care mitigated declines in in-person visits, its long-term health impacts remain unclear. COVID-19 created significant barriers to primary care access, and these findings may help decision-makers strengthen virtual care services to address reduced access during public health emergencies or workforce shortages. Monitoring primary care utilization trends allows governments and healthcare organizations to evaluate policies aimed at improving primary care access.
